# Beneficial effect of standardized extracts of *Amorphophallus paeoniifolius* tuber and its active constituents on experimental constipation in rats

**DOI:** 10.1016/j.heliyon.2020.e04023

**Published:** 2020-05-30

**Authors:** Yadu Nandan Dey, Manish M. Wanjari, Bhavana Srivastava, Dharmendra Kumar, Deepti Sharma, Jyoti Sharma, Sudesh Gaidhani

**Affiliations:** a School of Pharmaceutical Technology, Adamas University, Barasat, Kolkata, West Bengal, India; b Regional Ayurveda Research Institute for Drug Development, Gwalior, Madhya Pradesh, India; c Centre for Advanced Research in Pharmaceutical Sciences, Shobhit University, Meerut, Uttar Pradesh, India; d Faculty of Pharmaceutical Sciences, UCSI University, Kuala Lumpur, Malaysia; e Central Council for Research in Ayurvedic Sciences, New Delhi, India

**Keywords:** Food science, Pharmaceutical science, Loperamide, Rats, Constipation, Glucomannan, Betulinic acid, HPTLC

## Abstract

The tubers of *Amorphophallus paeoniifolius* (Elephant foot yam), principally consumed as crop food and vegetables, are used in ethno-medicinal practices in mitigation of constipation and piles. Hence, present study evaluated the effect of tubers of *A. paeoniifolius* and its active constituents glucomannan and betulinic acid on experimentally-induced constipation. The tuber and its extracts were standardized as per Ayurvedic Pharmacopoeia of India and physicochemical constants were found within the pharmacopoeial limit. HPTLC fingerprint profile of extracts has been developed using suitable mobile phase. Methanolic extract was subjected to column chromatography. The isolated phytoconstituents were characterized by FT-IR, NMR and MS and identified as betulinic acid and β-sitosterol. Functional constipation was induced in rats by oral administration of loperamide (3 mg/kg) for first 3 consecutive days. The rats were orally treated with methanolic and aqueous tuber extracts in the doses of 125, 250 and 500 mg/kg, glucomannan (300 mg/kg) and betulinic acid (1.5 mg/kg) for 7 days. The parameters viz. number of stools, wet weight of stools and moisture content of stools and intestinal transit were studied. Treatment with tuber extracts, glucomannan and betulinic acid showed significant (*p* < 0.05) increase in fecal parameters and intestinal transit in constipated rats. The effects were comparable to standard laxative drug, sodium picosulfate (5 mg/kg, orally). The results indicated that tuber extracts and its active constituents showed laxative effect and relieved constipation. It is concluded that tuber of *A. paeoniifolius* exhibited beneficial effect in functional constipation possibly through its laxative action. The study validates its ethno-medicinal use in correction of constipation. The principal constituents, betulinic acid and glucomannan in tuber extracts might have played important role in relieving the constipation.

## 1 Introduction

*Amorphophallus paeoniifolius* (Dennst.) Nicolson (family: Araceae) is an Ayurvedic medicinal plant useful for the treatment of gastrointestinal diseases viz. hemorrhoids, vomiting, anorexia, dyspepsia, flatulence, constipation, etc. (Anonymous, 2008; Nair, 1993). It is consumed by various tribes of India for mitigation of constipation, hemorrhoids and abdominal pain in ethnomedicinal practices (Rahman et al., 2013; Devi Prasad et al., 2013; Yesodharan and Sujana, 2007). Previous findings indicated that an oral administration of the tuber extracts in normal healthy rats produced significant increase in fecal output and the stimulation of intestinal motility (Dey et al., 2016). Several medicinal plants with prokinetic activity showed beneficial effect in constipation (Kakino et al., 2010; Muhammad et al., 2013). The content of glucomannan in the tuber extracts was also high (Dey et al., 2016). Further, we isolated betulinic acid and β-sitosterol from the tuber extract by column chromatography (Dey et al., 2017a), however, they were identified based on thin layer chromatography. Hence, the present study was conducted to characterize the isolated compounds of *A. paeoniifolius* tuber extracts and to investigate the effect of tuber extract and its constituents (glucomannan and betulinic acid) on experimental constipation on loperamide-induced constipation in rats and its modulation by standard laxative agent.

## 2 Experimental section

### 2.1 Plant material

The plant (*Amorphophallus paeoniifolius*) was purchased from the local market and authenticated at the Institute. A voucher specimen (No. 5-4/10-11/NRIASHRD/Tech/Survey/1611) was deposited in the herbarium of the Institute.

### 2.2 Standardization of crude powder of *Amorphophallus paeoniifolius* tuber

Physico-chemical paramaters like loss on drying at 105 °C, total ash, acid-insoluble ash, water soluble ash, alcohol soluble extractive, water-soluble extractive and pH of 10% aqueous solution of *A. paeoniifolius* tubers were determined using standard methods (Khandelwal, 2006). Fluorescence analysis was done by mixing the tuber powder of *A. paeoniifolius* in various reagents like HCl, H_2_SO_4,_ HNO_3_, FeCl_3_ and picric acid and seen in UV chamber (MAC, New Delhi) at 254 nm, 366 nm and visible light.

### 2.3 Extraction and HPTLC fingerprinting of extracts

Shade dried powder of tubers of *A. paeoniifolius* was subjected to Soxhlet extraction with methanol and further macerated in distilled water to get aqueous extract. The percent yield of reddish brown semisolid methanolic extract and brown solid aqueous extract were 9.48 % w/w and 6.16 % w/w, respectively.

For HPTLC studies 0. 5 g of methanolic extracts and 0.25 g of aqueous extract were weighed separately, dissolved in methanol and water, respectively to make 10 ml. Mobile phase used for developing the extracts was Chloroform:Methanol:Formic acid (6:4:0.2). Sampling of the extracts on the TLC plates was done by Linomat IV applicator of CAMAG HPTLC system. Ten and twenty microliters of methanolic extract were applied in track 1 and 2 whereas 10 and 20 μl of aqueous extract were applied in track 3 and 4 as 8 mm bands. The temperature and saturation time during the development were 27 ± 2 °C and 30 min, respectively. The developed plate was kept at 60 °C for 5 min and observed at 254 nm and 366 nm under UV cabinet. Further, the plate was derivatized in vanillin-sulfuric acid reagent followed by keeping at 105 °C till the appearance of band colors. The photographs and R_f_ values were documented by using Win CATS software. The HPTLC chromatograms were retrieved from Camag TLC Scanner.

### 2.4 Characterization of isolated phytoconstituents of methanolic extract of *Amorphophallus paeoniifolius tuber*

Methanolic extract (25 g) was eluted through column chromatography packed with silica gel (60–100 mesh) using mobile phase of pure benzene, benzene and ethyl acetate (19:1; 9:1; 4:1; 1:1) and pure ethyl acetate. Successive fractions were subjected to TLC. The fractions having similar TLC profile were combined and re-chromatographed till the isolation of pure compound. The isolated phyto-constituents were identified and characterized by ^1^H (Bruker AVLL-400 MHz FTNMR) and ^13^C NMR (Bruker AVLL-400 MHz FTNMR) and MS (JEOL-AccuTOF JMS-T100LC Mass spectrometer) spectral analysis.

### 2.5 Loperamide-induced constipation

#### 2.5.1 Animals

Healthy adult Wistar rats of either sex (220–250 g weight) were used for the study. Animals were housed as per the standard experimental conditions mentioned in our previous study (Dey et al., 2016). The experiments were done after the approval of the Institutional Animal Ethics Committee (Proposal No. NRIASHRD-GWL/IAEC/2013/01) of Regional Ayurveda Research Institute for Drug Development, Gwalior, India in accordance with guidelines of Committee for the Purpose of Control and Supervision of Experiments on Animals (CPCSEA), a Statutory Committee under Dept of Animal Husbandry and Dairying, Ministry of Fisheries, Animal Husbandry and Dairying, Government of India.

#### 2.5.2 Grouping, treatments and evaluation parameters

Animals were divided into 11 groups (6 rats/group). Methanolic and aqueous extract were suspended in 1% tween 80 as vehicle. Group I and II were designated as normal control (NC) and constipated vehicle control (CVC) rats which received distilled water and vehicle of the extracts, respectively. Group III rats were fed orally with standard laxative drug, sodium picosulfate (SPS, 5 mg/kg) (Méité et al., 2010). Groups IV-VI were fed orally with methanolic extract of tuber (125, 250 and 500 mg/kg) whereas groups VII-IX received orally aqueous extract of tuber (125, 250 and 500 mg/kg). Groups X-XI received orally glucomannan (300 mg/kg) (Widjanarko et al., 2013) and betulinic acid (1.5 mg/kg) (Onwuchekwa and Oluwole, 2015). Doses of methanolic and aqueous extracts were used as per the previous studies (Dey et al., 2016, 2017b). Functional constipation was induced in rats of group II-XI rats by oral administration of loperamide (3 mg/kg) for three consecutive days as described previously (Wintola et al., 2010). The drug treatments were continued co-jointly for seven consecutive days. The parameters used for assessing constipation were number, wet weight and moisture content of stools and intestinal transit as described previously (Yan et al., 2017). Briefly, each animal was kept individually in metabolic cage (Orchid Scientifics, Nasik, India) for four hours every day (10 am–2 pm). The number of stools was recorded, and collected wet stools were weighed and dried at 105 °C in hot air oven till constant weight was acquired. The moisture content of stools was calculated by following formula (Eq-1):

(1) $$\text{Moisture content}=\frac{\text{Wet weight of stools}-\text{Dry weight of stools }}{\text{Wet weight of stools}}\times 100$$

The average number, wet weight and moisture content of stools were calculated for each individual rat.

The rats were fasted overnight on day 7 of the treatment and intestinal transit was assessed on day 8 by measuring emptying of a non-nutrient solution. Each rat received a 1.5 ml test meal consisting of 0.05% phenol red in 1.5% aqueous methylcellulose solution by intragastric route. After 30 min, rats were sacrificed by a high dose of ether. The abdomen was cut opened and the intestine was mounted on a paper. The distance traveled by the phenol red meal in the intestine, from pylorus to cecum, was measured and expressed as percent intestinal transit (Yan et al., 2017) as described below (Eq-2).

(2) $$\text{\% Transit}=\frac{\text{Distance travelled by phenol red meal }}{\text{Total length of small intestine}}\times 100$$

### 2.6 Statistical analysis

All the data were analyzed with one-way ANOVA followed by Tukey's multiple comparison post hoc tests. A statistical difference of *p* < 0.05 was considered significant in all cases.

## 3 Results

### 3.1 Standardization of *Amorphophallus paeoniifolius* tuber

**Figure 1:**
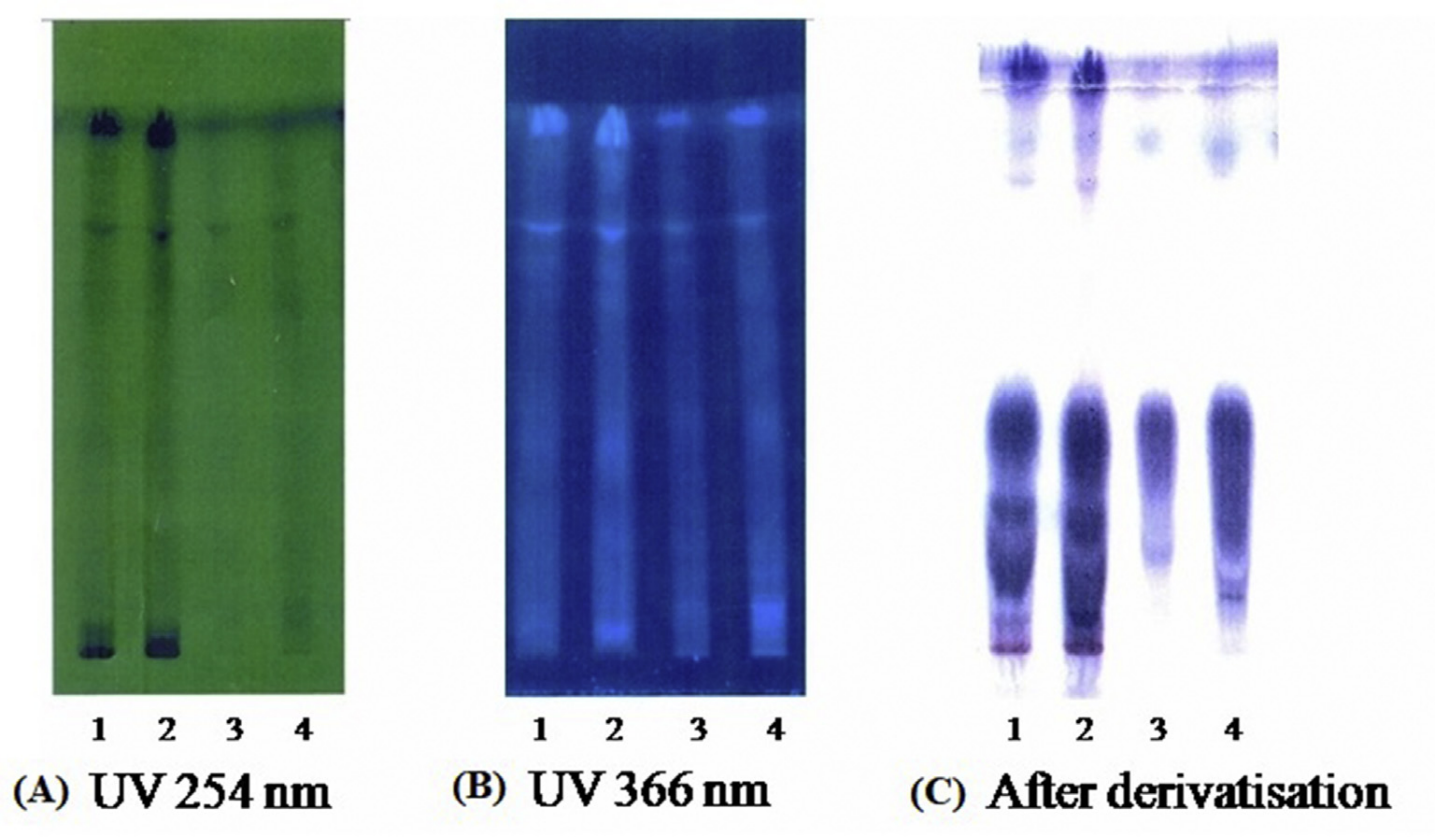
HPTLC chromatogram of *Amorphophallus paeoniifolius* tuber. (A) At UV 254 nm (B) At UV 366 nm (C) After derivatization. Track 1 (10 μg) and 2 (20 μg) of methanolic extract; Track 3 (10 μg) and 4 (10 μg) of aqueous extract. (For full non adjustable images, please refer Fig. S1 in supplementary material).

**Figure 2:**
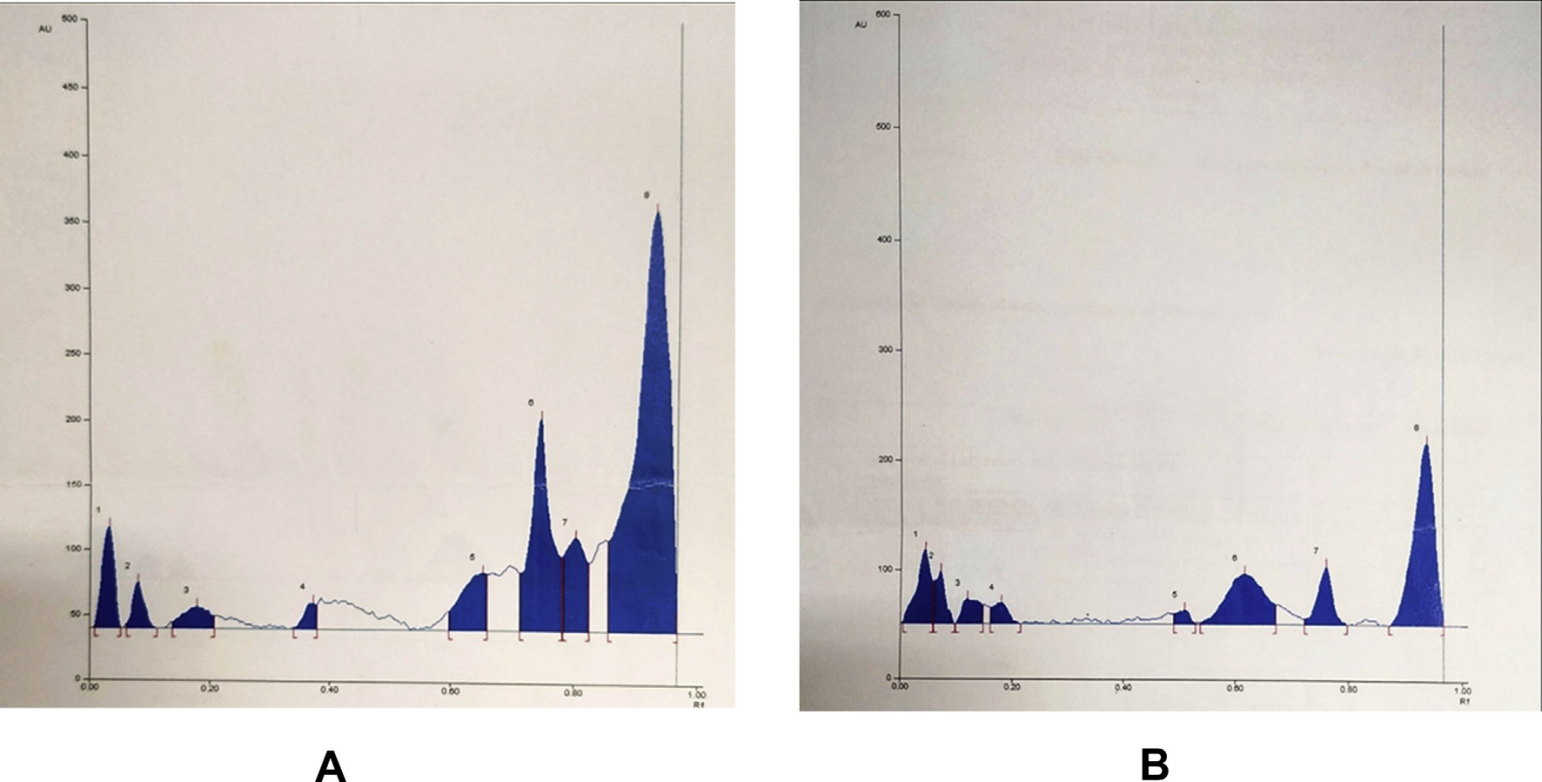
HPTLC densitogram of *Amorphophallus paeoniifolius* tuber. (A) Methanol extract (B) Aqueous extract (For full non adjustable images, please refer Fig. S2 and S3 in supplementary material).

The physico-chemical constants of tuber of *A. paeoniifolius* are mentioned in Table 1 which were well within the specified range and indicated that *A. paeoniifolius* tuber intended for study was of pharmacopoeial standard and up to the mark (Anonymous, 2008). HPTLC chromatograms and densitograms of methanol and aqueous extracts *of A. paeoniifolius* tuber are shown in Figures 1 and 2, respectively. For full non adjustable images, please refer to Fig. S1, S2 and S3 in supplementary material). The positions, heights and areas of different peaks are represented in Tables 2 and 3, respectively.

**Table 1:** Physico-chemical constants of tuber of *Amorphophallus paeoniifolius*.

Standardization parameters	Value
**Ash analysis (% w/w)**	
Ash content (Total ash)	4.35 ± 0.03
Acid in-soluble ash	0.44 ± 0.02
Water soluble ash	2.6 ± 0.21
**Extractive value (% w/w)**	
Water soluble	11.60 ± 0.21
Alcohol soluble	3.19 ± 0.04
**Moisture content (Loss on drying) (% w/w)**	1.08 ± 0.03
**pH (1% aqueous solution)**	5.49 ± 0.03

Values are expressed as mean ± SEM (n = 3).

**Table 2:** HPTLC profile of APME.

Peak	Start position (Rf)	Start Height (AU)	Max Position (Rf)	Max Height (AU)	Max %	End position (Rf)	End height (AU)	Area (AU)	Area %
1	0.01	1.2	0.01	78.6	10.53	0.05	0.9	1499.4	5.36
2	0.06	3.1	0.08	35.5	4.75	0.11	0.3	615.5	2.27
3	0.14	4.6	0.18	16.9	2.26	0.21	10.0	682.6	2.52
4	0.34	1.2	0.37	19.9	2.67	0.38	18.0	370.0	1.37
5	0.60	14.3	0.65	44.0	5.89	0.66	43.0	1690.7	6.25
6	0.71	44.2	0.75	162.4	21.74	0.78	56.8	4825.5	17.83
7	0.78	57.1	0.81	71.0	9.51	0.83	52.5	2216.4	8.19
8	0.86	68.9	0.93	318.5	42.65	0.97	16.7	15211.7	56.21

**Table 3:** HPTLC profile of APAE.

Peak	Start position (Rf)	Start Height (AU)	Max Position (Rf)	Max Height (AU)	Max %	End position (Rf)	End height (AU)	Area (AU)	Area %
1	0.00	1.2	0.04	67.2	15.44	0.06	38.5	1567.5	11.7
2	0.06	38.5	0.07	48.1	11.05	0.10	0.2	811.0	6.05
3	0.10	0.0	0.12	22.6	5.19	0.15	16.8	669.3	5.00
4	0.16	14.7	0.18	19.1	4.40	0.21	0.2	492.7	3.68
5	0.49	9.3	0.51	12.6	2.88	0.53	1.5	291.2	2.17
6	0.54	1.5	0.62	46.0	10.56	0.67	18.3	2895.1	21.61
7	0.72	5.5	0.76	53.3	12.30	0.80	0.1	1259.5	9.40
8	0.87	0.2	0.94	166.1	38.17	0.97	5.7	5408.1	40.08

### 3.2 Characterization of isolated compounds

**Figure 3:**
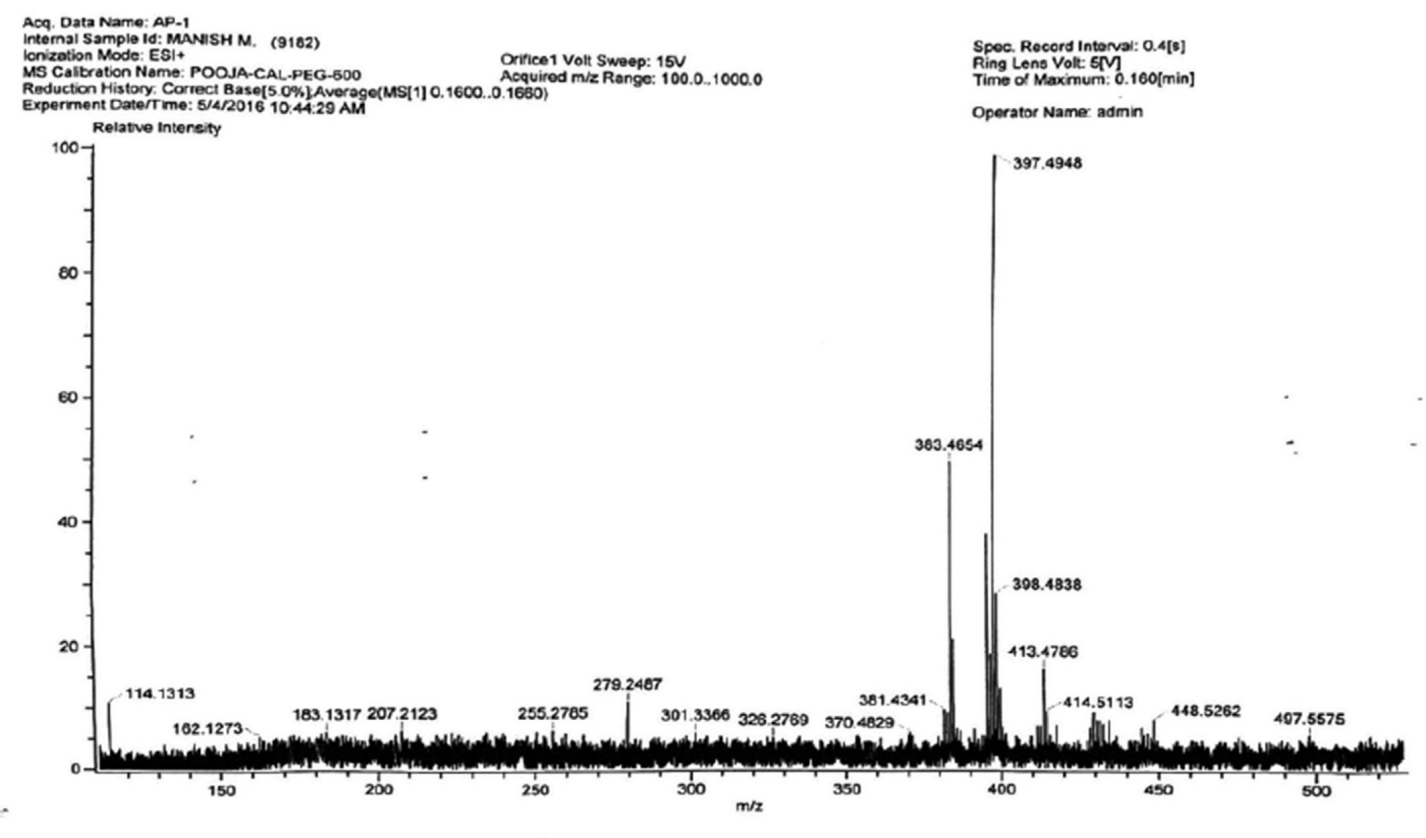
Mass spectrum of isolated compound 1. Mass spectrum of compound 1 showed m/z- 397.4948, m+ = 497.5575, (448.5262, 414.5113, 413.4786, 383.4654).

**Figure 4:**
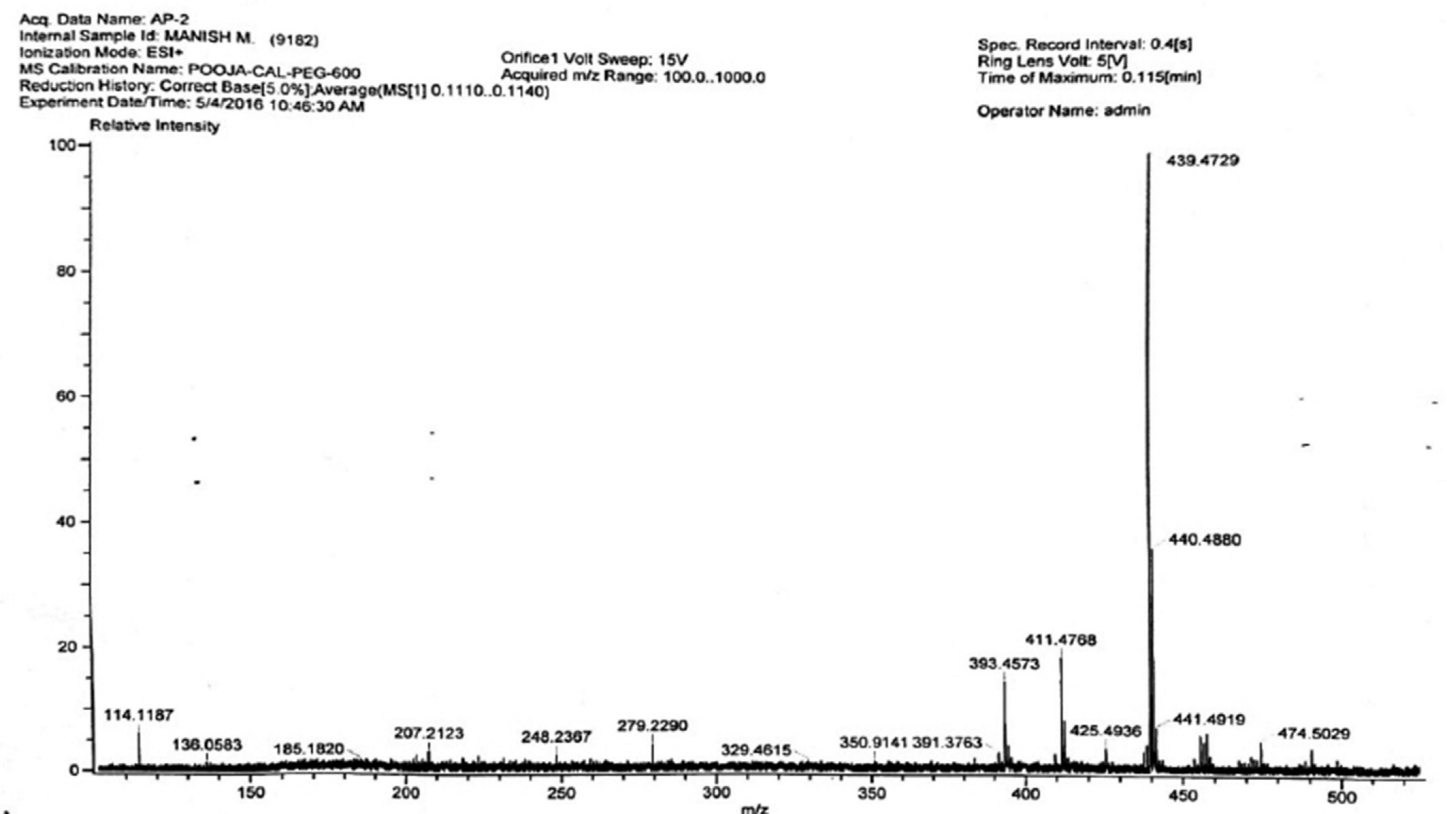
Mass spectrum of isolated compound 2. Mass spectrum of compound 2 showed m/z–439.4729, m+ = 474.5029, (441.4919, 411.4768, 3983.4573, 425.4936).

**Figure 5:**
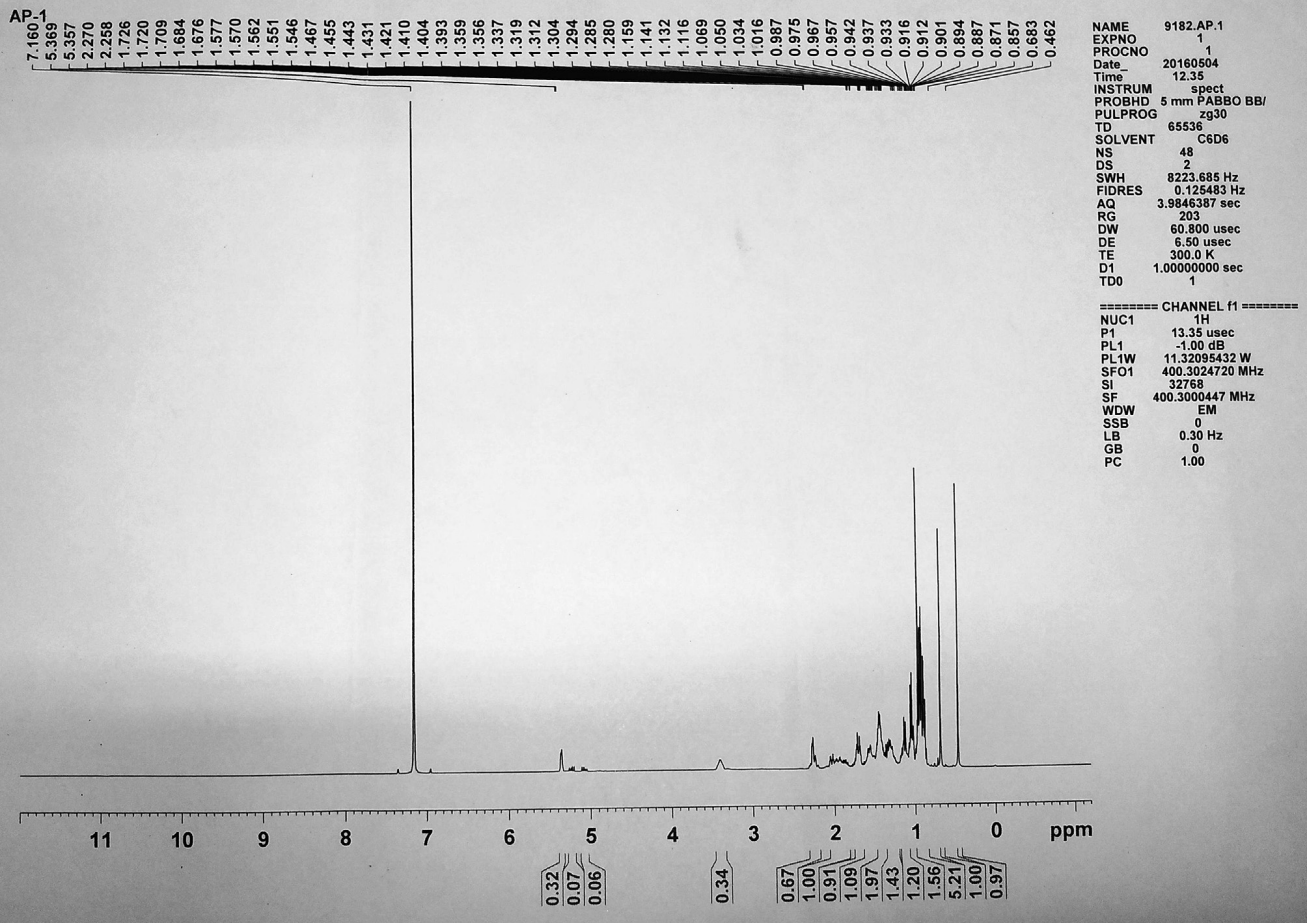
^1^H NMR spectrum of isolated compound 1.

**Figure 6:**
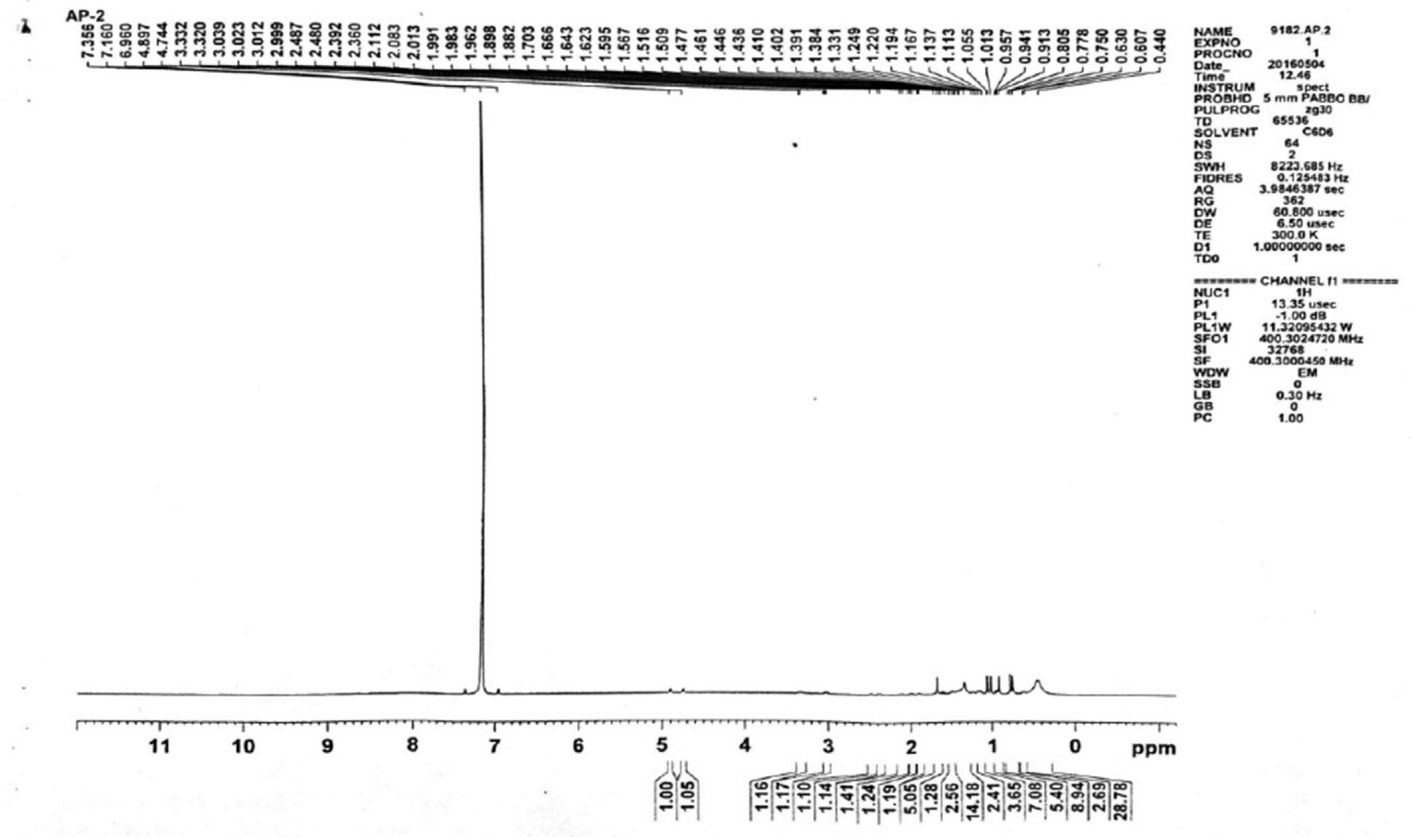
^1^H NMR spectrum of isolated compound 2.

**Figure 7:**
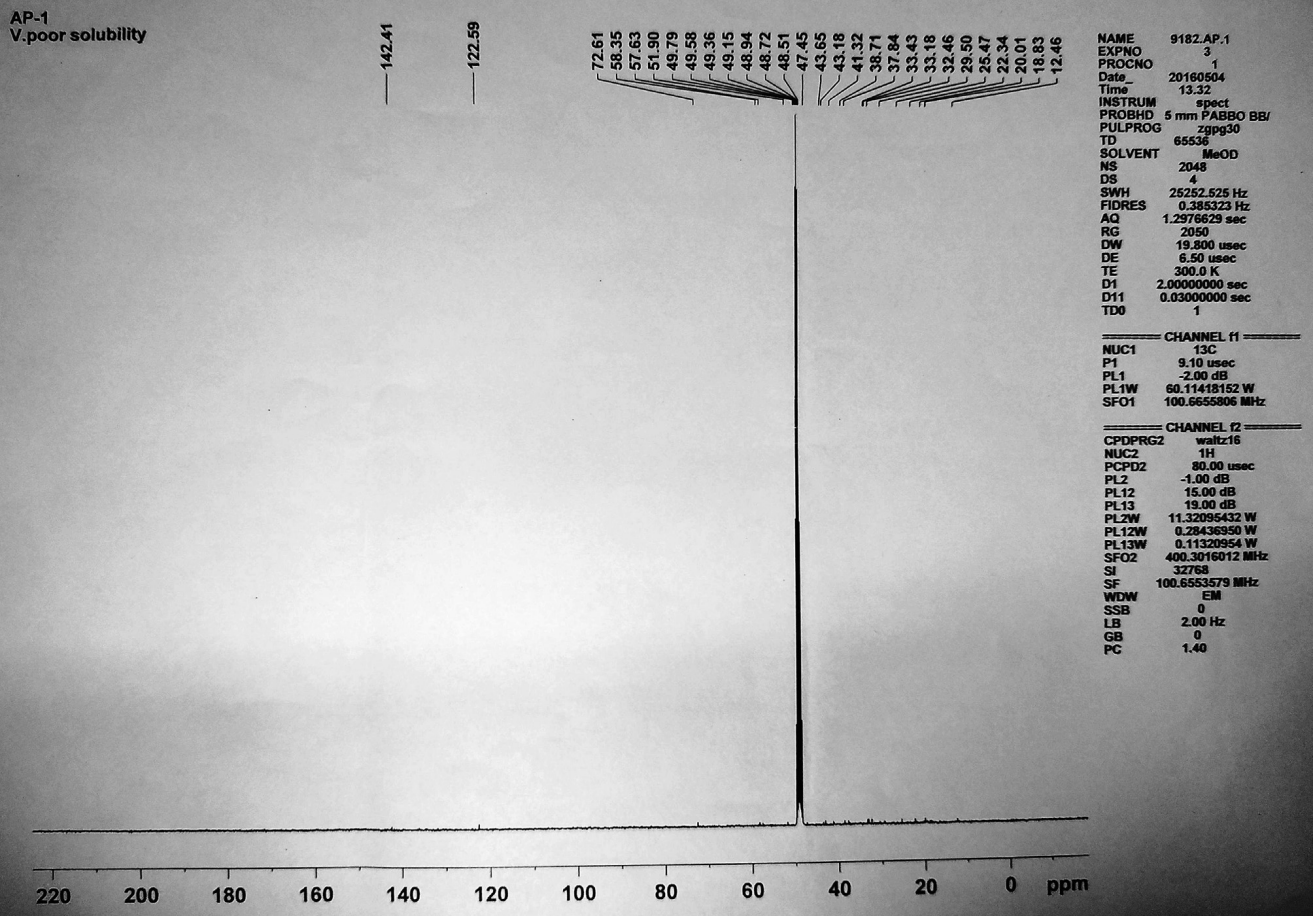
^13^C NMR spectrum of isolated compound 1.

**Figure 8:**
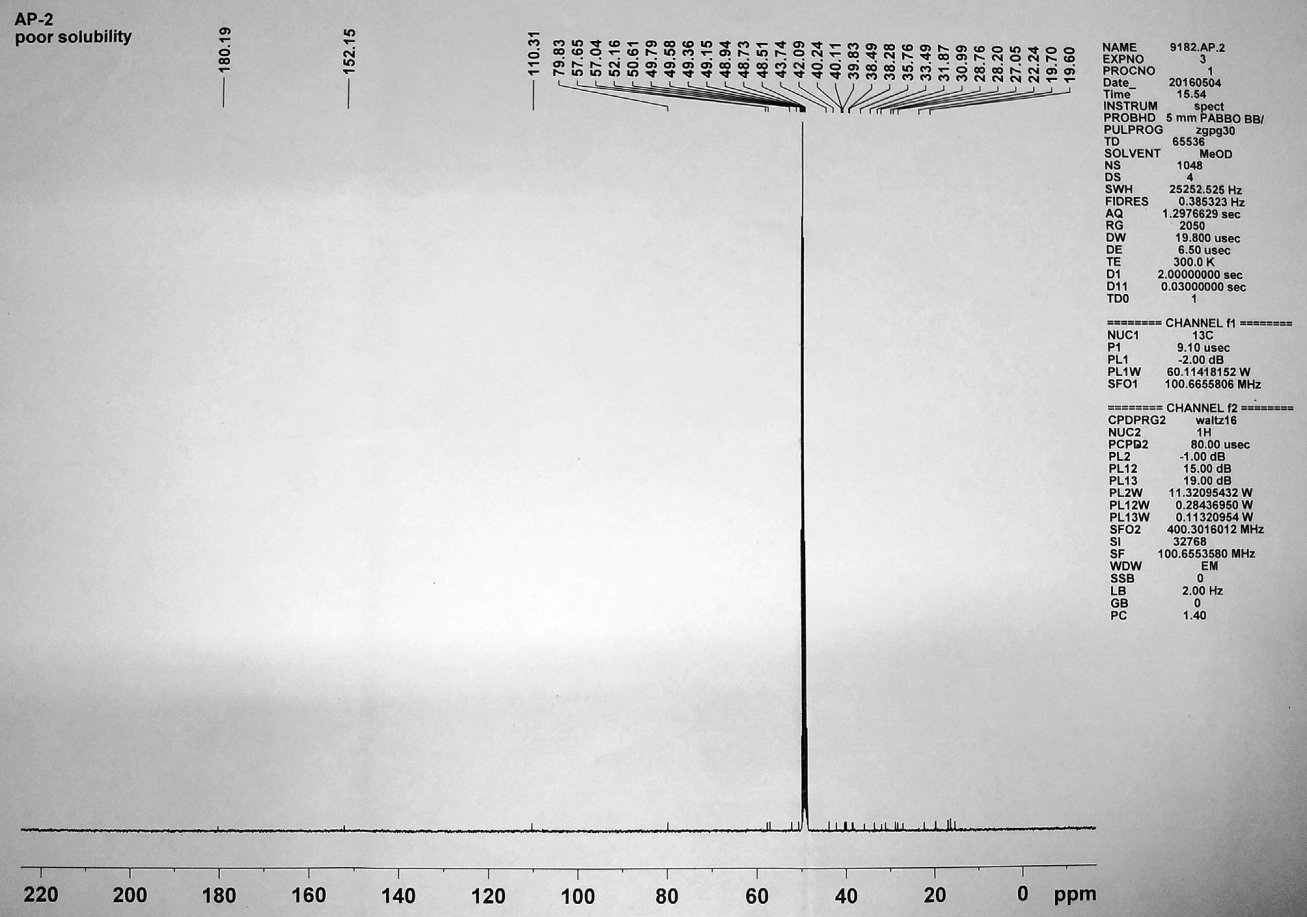
^13^C NMR spectrum of isolated compound 2.

Methanol extract of tuber *A. paeoniifolius* tuber yielded 2 phytoconstituents. The mass spectrum of compound 1 and 2 are dipicted in Figures 3 and 4. Mass spectrum confirms compound 1 as β-sitosterol whose MS-ion m/z 497.4, corresponding to protonated β-sitosterol dehydroxylation product [M−OH]^+^ (Mo et al., 2013). It also revealed compound 2 as betulinic acid whose MS-ion m/z 439.3 monitoring, corresponding to protonated betulinic acid dehydration product [M−H_2_O]^+^ (Şoica et al., 2012). The ^1^H and ^13^C NMR spectra of compound 1 and 2 are depicted in Figures 5, 6, 7, and 8. The detailed spectral assignments and their interpretations for compound 1 and 2 are described below.

#### 3.2.1 Identification of compounds

**Compound 1** (^1^H-NMR, CDCl_3_, ppm, 400 MHz): 5.35 (C=C–**H**, d, ^4^*J*_H-H_ = 4.8Hz, 1H), 3.39 (HO–C–**H**, t, *J* = 4.8 Hz, 1H), 2.26–1.01 (-CH_2_, m, 22H), 2.21(-OH, br-s, 1H), 1.05–1.70(tertiary-H, 7H), 1.01(-CH_3_, s, 3H), 0.91(-CH_3_, s, 3H), 0.93(-CH_3_, s, 3H), 0.88(-CH_3_, s, 3H), 0.68(-CH_3_, s, 3H), 0.46(-CH_3_, s, 3H). (^13^C-NMR, CDCl_3_, ppm, 100 MHz): 142.4 (-**C**=CH), 122.6 (-C=**C**H), 72.6(-**C**-OH), 58.4, 58.3, 57.6, 51.9, 47.4, 43.65, 43.18, 41.32, 38.71, 37.84, 35.10, 33.43, 33.18, 32.46, 30.10, 29.50, 26.30, 25.47, 22.34, 20.01, 19.80, 18.30, 18.60, 18.83, 16.50 and 12.46 (–CH_2_–**C**H_3_).

**Compound 2** (^1^H-NMR, CDCl_3_, ppm, 400 MHz): 4.89 (C=C**H**, s, 1H), 4.74(C=C**H**, s, 1H), 3.33(HO–C–**H**, td, ^3^*J*_syn_ = 11.2 Hz, ^3^*J*_anti_ = 4.8 Hz, 1H), 3.02 (H_2_C = C–C–**H**, 1H), 1.22–2.01(tertiary-H, 4H), 2.50–1.22 (-CH_2_, m, 20H), 1.88(-OH, br-s, 1H), 1.70(-CH_3_, s, 3H), 1.33(-CH_3_, s, 3H), 1.05(-CH_3_, s, 3H), 1.19(-CH_3_, s, 3H), 0.90(-CH_3_, s, 3H), 0.75(-CH_3_, s, 3H). (^13^C-NMR, CDCl_3_, ppm, 100 MHz): 180.19 (-**C**OOH), 152.15 (-**C**=CH_2_), 110.31 (-C=**C**H_2_), 79.83 (**C**–OH), 57.65, 57.04, 52.16, 50.61, 43.74, 42.09, 40.24, 40.11, 39.83, 38.49, 38.28, 35.76, 33.49, 31.87, 30.99, 28.76, 28.20, 27.05, 22.24, 19.70, 19.60, 16.87, 16.79, 16.25, 15.25.

#### 3.2.2 Interpreatation of spectral data of compound 1

In ^1^H-NMR, the presence of 6 signals at 1.01, 0.91, 0.93, 0.88, 0.68, and 0.46 ppm integrating to 3 protons each confirms the 6-CH_3_ groups. Proton attached to the alkene moiety resonates as a doublet at 5.35 ppm with ^4^*J*_H-H_ = 4.8 Hz. A triplet at 3.39 ppm with ^3^*J*_H-H_ = 4.8 Hz corresponds to the proton attached to the carbon having OH group. A broad singlet observed at 2.21 ppm can be attributed to the proton of –OH group. The 22 diastereotopic methylene protons resonated as multiplets in the range 2.26-1.01 ppm whereas the other 7 protons attached to the tertiary carbons resonated in the range 1.05–1.70 as multiplets.

In ^13^C-NMR, the alkene carbons appeared in the most downfield region at 142.4 and 122.6 ppm. The carbon attached to the –OH group appeared at 72.6 ppm. Rest other carbons resonated in the range 58.4-12.4 ppm at their expected region.

#### 3.2.3 Interpreatation of spectral data of compound 2

In ^1^H-NMR, the presence of 6 signals at 1.70, 1.33, 1.05, 1.19, 0.90 and 0.75 ppm integrating to 3 protons each confirms the 6-CH_3_ groups. Methylene Proton attached resonated as a singlet at 4.89 and 4.74 ppm. A triplet of doublet at 3.33 ppm with ^3^*J*_syn_ = 11.2 Hz and ^3^*J*_anti_ = 4.8 Hz corresponds to the proton attached to the carbon having OH group. The proton attached to the carbon having the alkyl substituent in the five membered ring resonated at 3.02 ppm as a multiplet. A broad singlet observed at 1.88 ppm can be attributed to the proton of –OH group. The 20 diastereotopic methylene protons resonated as multiplets in the range 2.50-1.22 ppm whereas the other 4 protons attached to the tertiary carbons resonated in the range 1.22–2.01 as multiplets.

In ^13^C-NMR, the –COOH carbon appeared in the most downfield region at 180.19 ppm. The alkene carbons appeared at 152.15 and 110.31 ppm. The carbon attached to the –OH group appeared at 79.83 ppm. Rest other carbons resonated in the range 57.65-15.25 ppm at their expected region.

By comparing the spectral data with the previous literatures (Garcez et al., 2003; Ayatollahi et al., 2011), compound 1 and 2 were found to be β-sitosterol and betulinic acid, respectively.

### 3.3 Effect on functional constipation

#### 3.3.1 Number of stools

**Figure 9:**
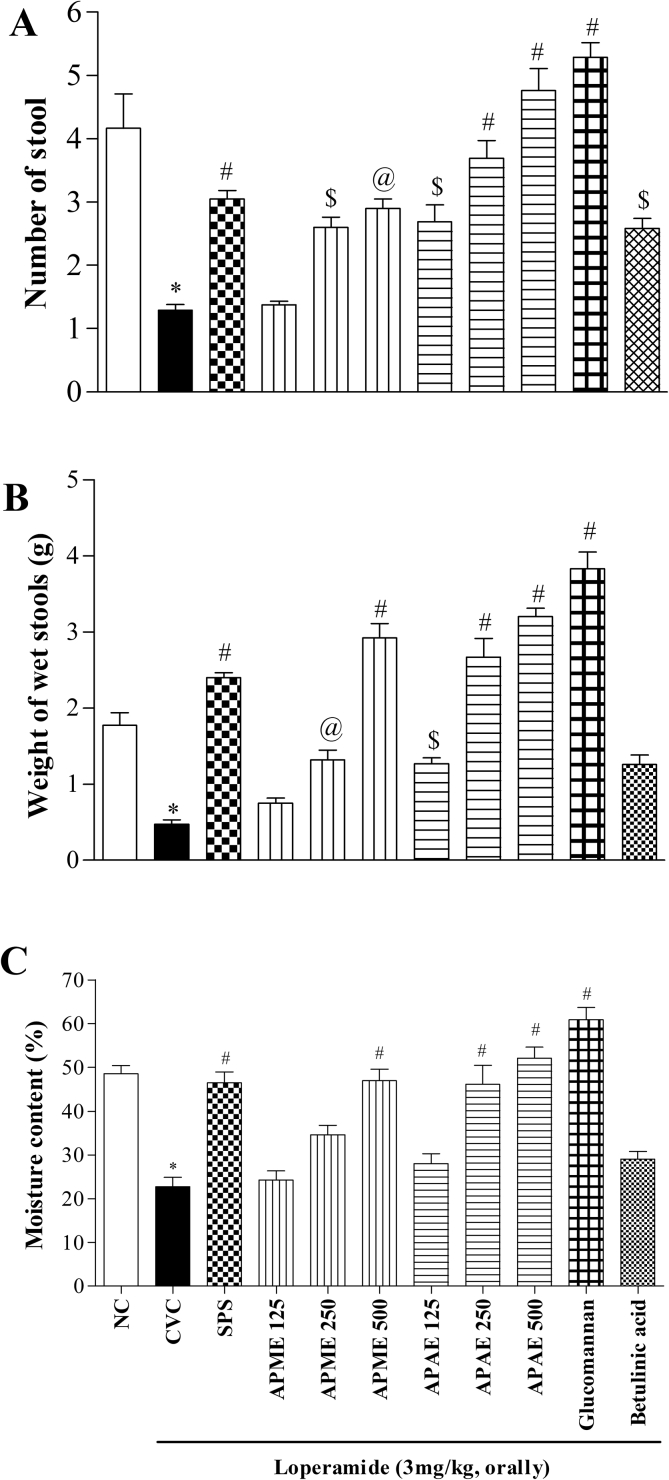
Effect on fecal parameters (A) Number of stool (B) Weight of wet stools (C) Moisture content. Results are expressed as mean ± SEM (N = 6) ∗*p* < 0.001 compared to NC; $*p* < 0.05, @*p* < 0.01, #*p* < 0.001 compared to CVC. [NC-Normal control, CVC-Constipated vehicle control, SPS- sodium picosulfate, Methanolic (APME) and aqueous (APAE) extract of *Amorphophallus paeoniifolius*]. Doses are expressed as mg/kg.

Administration of loperamide (3 mg/kg, orally) caused significant decrease in the number of stools, wet weight of stools and moisture content of stools when compared to NC rats (*p* < 0.001). Treatment with methanolic extract (250 and 500 mg/kg) and aqueous extract (125, 250 and 500 mg/kg) significantly (*p* < 0.05 to *p* < 0.001, wherever applicable) increased the number of stools compared to CVC group. Standard drug, SPS (5 mg/kg) also showed significant (*p* < 0.001) increase in number of stools compared to CVC group (Figure 9A). Similarly, treatment with glucomannan (300 mg/kg) and betulinic acid (1.5 mg/kg) significantly (*p* < 0.5 and *p* < 0.001) increased number of stools compared to CVC group (Figure 9A).

#### 3.3.2 Effect on wet weight of stools

Administration of loperamide (3 mg/kg, orally) significantly decreased wet weight of stools compared to NC rats (*p* < 0.001). Treatment with methanolic extract (250 and 500 mg/kg) and aqueous extract (125, 250 and 500 mg/kg) significantly (*p* < 0.05 to *p* < 0.001, wherever applicable) increased the wet weight of stools compared to CVC group similar to standard drug, SPS (5 mg/kg) (*p* < 0.001) (Figure 9B). Treatment with glucomannan (300 mg/kg) and betulinic acid (1.5 mg/kg) also showed significant (*p* < 0.05 and *p* < 0.001) increase in wet weight of stools compared to CVC group (Figure 9B).

#### 3.3.3 Effect on moisture content of stools

Administration of loperamide (3 mg/kg, orally) caused significant decrease in moisture content of stools when compared to NC rats (*p* < 0.001). Treatment with methanolic extract (500 mg/kg) and aqueous extract (250 and 500 mg/kg) showed significant (*p* < 0.01 to *p* < 0.001, wherever applicable) increase in moisture content of stools compared to CVC rats. The standard drug, SPS (5 mg/kg) also significantly (*p* < 0.001) increased moisture content of stools compared to CVC group (Figure 9C). Glucomannan (300 mg/kg) treatment showed significant (*p* < 0.001) increase in moisture content of stools whereas betulinic acid (1.5 mg/kg) did not show any significant changes compared to CVC rats (Figure 9C).

#### 3.3.4 Effect on intestinal transit

**Figure 10:**
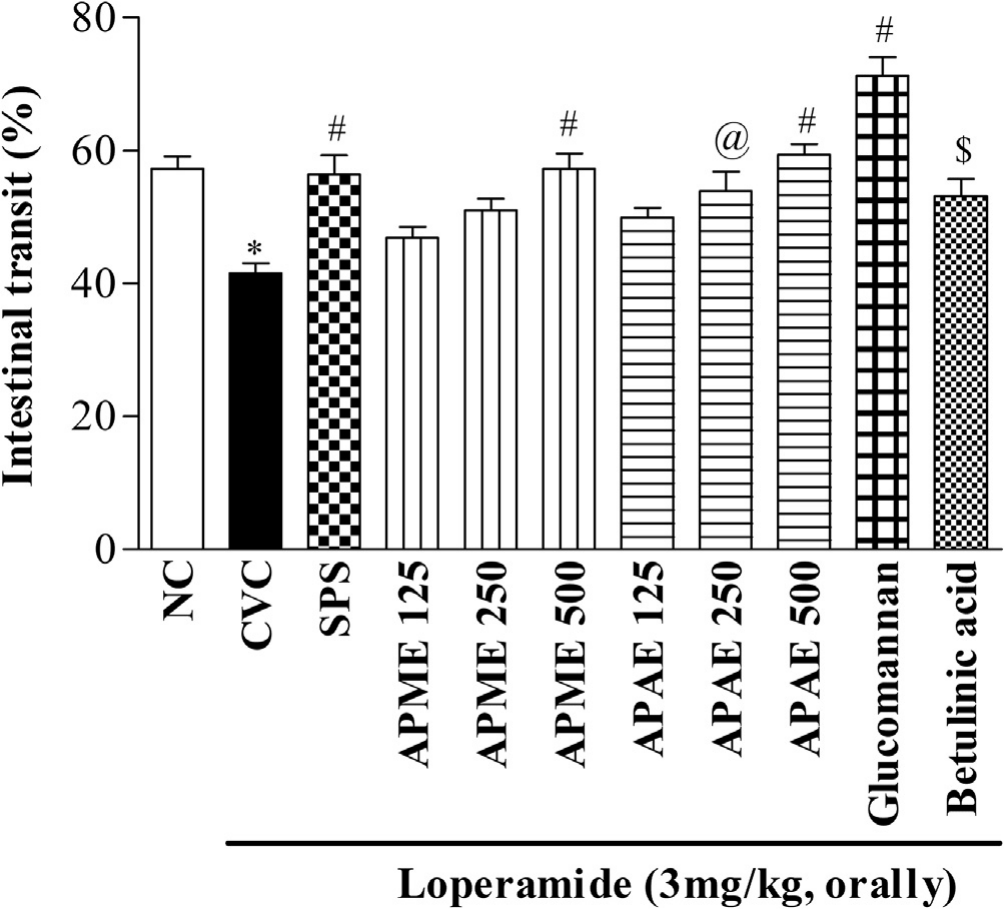
Effect on intestinal transit. Results are expressed as mean ± SEM (N = 6) ∗*p* < 0.001 compared to NC; $*p* < 0.05, @*p* < 0.01, #*p* < 0.001 compared to constipated vehicle control (CVC). [NC-Normal control, CVC-Constipated vehicle control, SPS- sodium picosulfate, Methanolic (APME) and aqueous (APAE) extract of *Amorphophallus paeoniifolius*]. Doses are expressed as mg/kg.

Administration of loperamide (3 mg/kg) caused significant decrease in intestinal transit when compared to NC rats (*p* < 0.001). Treatment with methanol and aqueous tuber extracts (250 and 500 mg/kg) (*p* < 0.05) (Figure 10) showed dose dependent increase in intestinal transit as compared to CVC group. The standard drug, SPS (5 mg/kg) also showed similar increasing (*p* < 0.001) effect. Treatment with glucomannan (300 mg/kg) and betulinic acid (1.5 mg/kg) significantly (*p* < 0.001 and *p* < 0.05, respectively) increased the intestinal transit compared to CVC rats (Figure 10).

## 4 Discussion

In the present study, the tuber extracts of *A. paeoniifolius* demonstrated amelioration of loperamide-induced functional constipation in rats. The tubers used for the present study were first standardized as per pharmacopoeial standards using physicochemical constants viz. various ash contents, extractive values, loss on drying and pH which are considered very important parameters for assessing the quality of herbal raw material. The higher ash value is an indication of drug adulteration or inappropriate processing of the raw material and any marked difference indicates a change in quality. The quality of the raw material can be judged from extractive value determination. This is because already exhausted raw material will result in lower extractive values. In the present study, physicochemical constants viz. ash content, extractive value, LOD and pH were well within the specified range indicated that *A. paeoniifolius* tuber intended for study was of pharmacopoeial standard and up to the mark (Anonymous, 2008).

The phytochemical screening and quantitative estimation of various phytochemicals are helpful for proper standardization of the herbal drug for its various pharmacological potentials and checking for any variation. The fingerprints from HPTLC and HPLC are very useful for qualitative and quantitative analysis of herbal drug formulations. The chromatograms shown in Figure 2 A and B indicate that maximum constituents of methanolic extract and aqueous extracts were clearly separated without tailing and diffuseness. It is evident from Table 2 that in 20 μl of methanolic extract (track 2) there are 8 bands indicating the occurrence of at least 08 different components in this extract. Out of these 8 components, the component with R_f_ values 0.75 and 0.93 were found to be more predominant as the percentage area of these bands were more with 17.83% and 56.21 % respectively. The remaining components were found to be very less in quantity as the percent area for all these bands were less than 10 %. In 20 μl of aqueous extract (track 4) there are again 8 bands (Table 3) indicating the occurrence of at least 08 different components. Out of these 08 components, the component with R_f_ values 0.04, 0.62 and 0.94 were more predominant as the percentage area of these bands were more with 11.70%, 21.61 % and 40.38%, respectively. The remaining components were found to be very less in quantity as the percent area for all the bands were less than 10 %.

Isolation of the methanolic extract by column chromatography revealed two isolated compounds - compound 1 and compound 2. The phytochemical and physical interpretation of spectral data obtained from the two isolated compounds β-sitosterol and betulinic acid. Previous literature also suggested the presence of these phytochemicals in the tuber (Srivastava et al., 2014; Tandon and Sharma, 2013). The chromatographic fingerprints as well as isolation of two compounds i.e. β-sitosterol and betulinic acid of the tuber of *A. paeoniifolius* revealed the phytochemical composition of the tuber extracts.

Pharmacological study revealed that administration of loperamide significantly decreased the number of stools, wet weight and moisture content of stools, and intestinal transit when compared to normal control rats which confirmed the induction of constipation. Loperamide induces constipation by decreasing intestinal fluid secretion and intestinal motility which leads to delayed fecal evacuation and intestinal transit (Holzer, 2009). Dose dependant increase in number of stools and wet weight and moisture content of stools and intestinal transit by treatment with methanol and aqueous extract indicates amelioration of constipation and suggests laxative effect of the drug. The results were comparable to standard laxative drug, sodium picosulfate and support the findings. The increased wet weight and moisture content of stools indicates increase in water portion in stools which might have loosened the stool consistency and facilitated the easy propulsion. The dietary fibers affect gastrointestinal transit time and bulkiness of stools by increasing the water content and their bacterial degradation (Stevens et al., 1988). The tuber contains nearly 70% of carbohydrates, and has high glucomannan, water-soluble fiber content (Srivastava et al., 2014, Nguyen et al., 2009). Glucomannan has bulk forming action and increases the stool volume and bulkiness of stool (Tungland and Meyer, 2002). Phytochemical studies in our previous study estimated high glucomannan content in *A. paeoniifolius* tuber extracts. The methanolic and aqueous extract of *A. paeoniifolius* tuber contain 1.13% and 9.04 % glucomannan, respectively (Dey et al., 2016). In the present study, treatment of glucomannan (300 mg/kg) caused attenuation of fecal parameters and intestinal transit which confirms and attributes the role of glucomannan in observed increase in stool number and weight, and moisture content by methanolic and aqueous extracts of *A. paeoniifolius*. Glucomannan is reported to increase the frequency of stools and relieves constipation in children (Loening-Baucke et al., 2004). This supports the present findings. Previously, many dietary fibers showed laxative action by increasing the fecal content and moisture content as well as intestinal transit and strengthens the findings (Tungland and Meyer, 2002).

Loperamide caused significant decrease in intestinal transit compared to normal control rats which indicates the inhibition of gastrointestinal motor function. Treatment with tuber extracts exhibited increase in intestinal transit when compared to vehicle control rats which indicates the enhanced gastrointestinal motility and gastrointestinal motor function and suggests the spasmogenic action of the extracts. In our previous study, tuber extracts showed enhancement in gastrointestinal motility as observed by increased fundus and ileum contractility (Dey et al., 2016). The spasmogenic effect of the extract might be playing a major role in reversal of constipation. Betulinic acid showed spamsogenic effect by partial agonistic action in the serotonergic (5HT) receptors on rat fundus preparation (Bejar et al., 1995). In the present study, the treatment of betulinic acid caused significant attenuation of intestinal transit compared to constipated vehicle control group. It also showed laxative effect as indicated by significant inhibition of increase in stool number and weight. However, betulinic acid did not significantly affect the moisture content of feces which indicates that the observed laxative effect might be due to the spasmogenic activity only. Glucomannan was also reported to have influence on 5-HT in gastrointestinal tract and results in contraction It is fermented and degraded by colonic bacteria to short chain fatty acids which cause increase in serotonin levels leading to enhanced colonic motility (Widjanarko et al., 2013). However, there is no report of action of glucomannan and betulinic acid on specific serotonergic receptors. The present study also opens up scope to explore the role of specific serotonergic receptors involved in anticonstipating/prokinetic action of glucomannan and betulinic acid. Thus, motility enhancing activity of the extracts may be due to betulinic acid and glucomannan possibly through spasmogenic effect. However, further studies are required to identify other major phytoconstituents and elucidate the exact mechanism involved along with role of 5-HT receptors in laxative action of *A. paeoniifolius* tuber.

Current study demonstrated the influence of tuber extracts on loperamide-induced constipation in rats which is the first attempt to evaluate the effect of *A. paeoniifolius* tuber in constipated rats. Results revealed that both methanolic and aqueous extracts of *A. paeoniifolius* tuber were found effective in amelioration of constipation. However, the aqueous extract caused more significant attenuation of the fecal parameters than methanolic extract. The study also evaluated the effect of major constituent of *A. paeoniifolis* tuber in loperamide-induced constipation in rats which identified the major bioactive constituents of *A. paeoniifolius* tuber in relieving constipation.

## 5 Conclusion

In conclusion, the tuber of *A. paeoniifolius* and its active constituents (glucomannan and betulinic acid) exhibited beneficial effect on loperamide-induced constipation in rats. This substantiates its traditional and ethnomedicinal use in correction of constipation. The laxative action of the tuber extracts implicates its therapeutic effect in treatment of hemorrhoids/piles, an associated disorder of constipation.

## Declarations

### Author contribution statement

Yadu N. Dey: Conceived and designed the experiments; Performed the experiments; Analyzed and interpreted the data; Contributed reagents, materials, analysis tools or data; Wrote the paper.

Manish M. Wanjari: Conceived and designed the experiments; Analyzed and interpreted the data; Contributed reagents, materials, analysis tools or data; Wrote the paper.

Bhavana Srivastava: Analyzed and interpreted the data.

Dharmendra Kumar, Jyoti Sharma, Sudesh Gaidhani: Contributed reagents, materials, analysis tools or data.

Deepti Sharma: Performed the experiments.

### Funding statement

This research did not receive any specific grant from funding agencies in the public, commercial, or not-for-profit sectors.

### Competing interest statement

The authors declare no conflict of interest.

### Additional information

No additional information is available for this paper.

## Appendix A Supplementary data

Supplementary materials.docx

## References

[bib1] Anonymous (2008).

[bib2] Ayatollahi A.M., Ghanadian M., Afsharypour S., Abdella O.M., Mirzai M., Askari G. (2011). Pentacyclic triterpenes in *Euphorbia microsciadia w*ith their T-cell proliferation activity. Iran. J. Pharm. Res..

[bib3] Bejar E., Amarquaye A., Che C.T., Malone M.H., Fong H.H. (1995). Constituents of *Byrsonima crassifolia* and their spasmogenic activity. Int. J. Pharmacogn..

[bib4] Devi Prasad A.G., Shyma T.B., Raghavendra M.P. (2013). Plants used by the tribes for the treatment of digestive system disorders in Wayanad district, Kerala. J. Appl. Pharmaceut. Sci..

[bib5] Dey Y.N., Mahor S., Kumar D., Wanjari M., Gaidhani S., Jadhav A. (2016). Gastrokinetic activity of *Amorphophallus paeoniifolius* tuber in rats. J. Intercult. Ethnopharmacol..

[bib6] Dey Y.N., Sharma G., Wanjari M.M., Kumar D., Lomash V., Jadhav A.D. (2017). Beneficial effect of *Amorphophallus paeoniifolius* tuber on experimental ulcerative colitis in rats. Pharmaceut. Biol..

[bib7] Dey Y.N., Wanjari M.M., Kumar D., Lomash V., Gaidhani S.N., Jadhav A.D. (2017). Oral toxicity of elephant foot yam (*Amorphophallus paeoniifolius*) tuber in mice. J. Pharm. Pharmacogn. Res..

[bib8] Garcez F.R., Garcez W.S., Miguel D.L.S., Serea A.T., Prado F.C. (2003). Chemical constituents from *Terminalia glabrescens*. J. Braz. Chem. Soc..

[bib9] Holzer P. (2009). Opioid receptors in the gastrointestinal tract. Regul. Pept..

[bib10] Kakino M., Izuta H., Ito T., Tsuruma K., Araki Y., Shimazawa M., Oyama M., Iinuma M., Hara H. (2010). Agarwood induced laxative effects via acetylcholine receptors on loperamide-induced constipation in mice. Biosci. Biotechnol. Biochem..

[bib11] Khandelwal K.R. (2006). Practical Pharmacognosy.

[bib13] Loening-Baucke V., Miele E., Staiano A. (2004). Fiber (glucomannan) is beneficial in the treatment of childhood constipation. Pediatrics.

[bib14] Méité S., Bahi C., Yéo D., Datté J.Y., Djaman J.A., N’guessan D.J. (2010). Laxative activities of *Mareya micrantha* (Benth.) Müll. Arg. (Euphorbiaceae) leaf aqueous extract in rats. BMC Complement. Altern. Med..

[bib15] Mo S., Dong L., Hurst W.J., Breemen R.B.V. (2013). Quantitative analysis of phytosterols in edible oils using APCI liquid chromatography-tandem mass spectrometry. Lipids.

[bib16] Muhammad N., Rehman N.U., Khan H., Saeed M., Gilani H.S. (2013). Prokinetic and laxative effects of the crude methanolic extract of *Viola betonicifolia* whole plant in rodents. BMC Comp. Alt. Med..

[bib17] Nair R.V. (1993).

[bib18] Nguyen T.A., Do T.T., Nguyen T.D., Pham L.D., Nguyen V.D. (2009). Characterization of polysaccharide from *Amorphophallus paeoniifolius* in Vietnam. J. Chem..

[bib19] Onwuchekwa C., Oluwole F.S. (2015). Anti-gastric ulcer effect of betulinic acid in male albino rats. Niger. J. Physiol. Sci..

[bib20] Rahman A.H.M.M., Nitu S.K., Ferdows Z., Islam A.K.M.R. (2013). Medico-botany on herbaceous plants of Rajshahi, Banglabesh. Am. J. Life Sci..

[bib21] Şoica C.M., Dehelean C.A., Peev C., Aluas M., Zupkó I., Kása P., Alexa E. (2012). Physico-chemical comparison of betulinic acid, betulin and birch bark extract and *in vitro* investigation of their cytotoxic effects towards skin epidermoid carcinoma (A431), breast carcinoma (MCF7) and cervix adenocarcinoma (HeLa) cell lines. Nat. Prod. Res..

[bib22] Srivastava S., Verma D., Srivastava A., Tiwari S.S., Dixit B. (2014). Phytochemical and nutritional evaluation of *Amorphophallus campanulatus* (Roxb.) Blume Corm. J. Nutr. Food Sci..

[bib23] Stevens J., VanSoest P.J., Robertson J.B., Levitsky D.A. (1988). Comparison of the effects of psyllium and wheat bran on gastrointestinal transit time and stool characteristics. J. Am. Diet Assoc..

[bib24] Tandon N., Sharma P. (2013).

[bib25] Tungland B.C., Meyer D. (2002). Nondigestible oligo- and polysaccharides (dietary fiber):Their physiology and role in human health and food. Compr. Rev. Food Sci. Food Saf..

[bib26] Widjanarko S.M., Wijayanti N., Sutrisno A. (2013). Laxative potential of the konjac flour (*Amorphophallus muelleri* Blume) in treatment of loperamide induced constipation on Sprague Dawley rats. Int. J. Med. Health Sci..

[bib27] Wintola O.A., Sunmonu T.O., Afolayan A.J. (2010). The effect of *Aloe ferox* Mill. in the treatment of loperamide-induced constipation in Wistar rats. BMC Gastroenterol..

[bib28] Yan S., Yue Y.Z., Wang X.P., Dong H.L., Zhen S.G., Wu B.S., Qian H.H. (2017). Aqueous extracts of *Herba cistanche* promoted intestinal motility in loperamide-induced constipation rats by ameliorating the interstitial cells of cajal. Evid. Based Compl. Alt. Med..

[bib29] Yesodharan K., Sujana K.A. (2007). Wild edible plants traditionally used by the tribes in the Parambikulam Wildlife Sanctuary, Kerala, India. Nat. Prod. Rad..

