# Supplementary material for: Beneficial effect of standardized extracts of Amorphophallus paeoniifolius tuber and its active constituents on experimental constipation in rats
Source: Heliyon. 2020 May 30;6(5):e04023. doi: 10.1016/j.heliyon.2020.e04023 (PMC7264754; doi:10.1016/j.heliyon.2020.e04023)
Supplement: Supplementary file 1 — Supplementary materials.docx [file mmc1.docx]

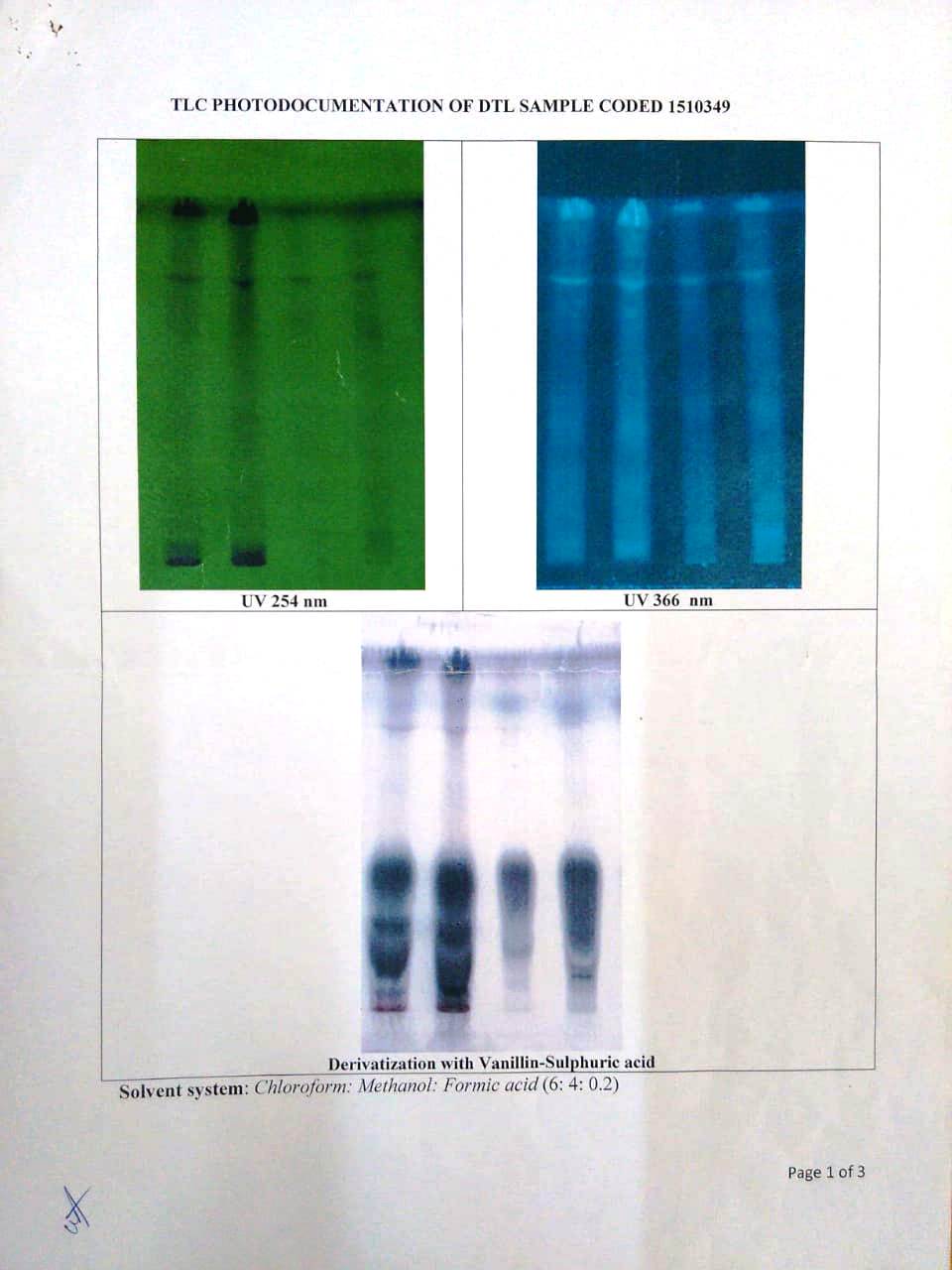


**Fig. S1.** **HPTLC chromatogram of *Amorphophallus paeoniifolius* tuber**


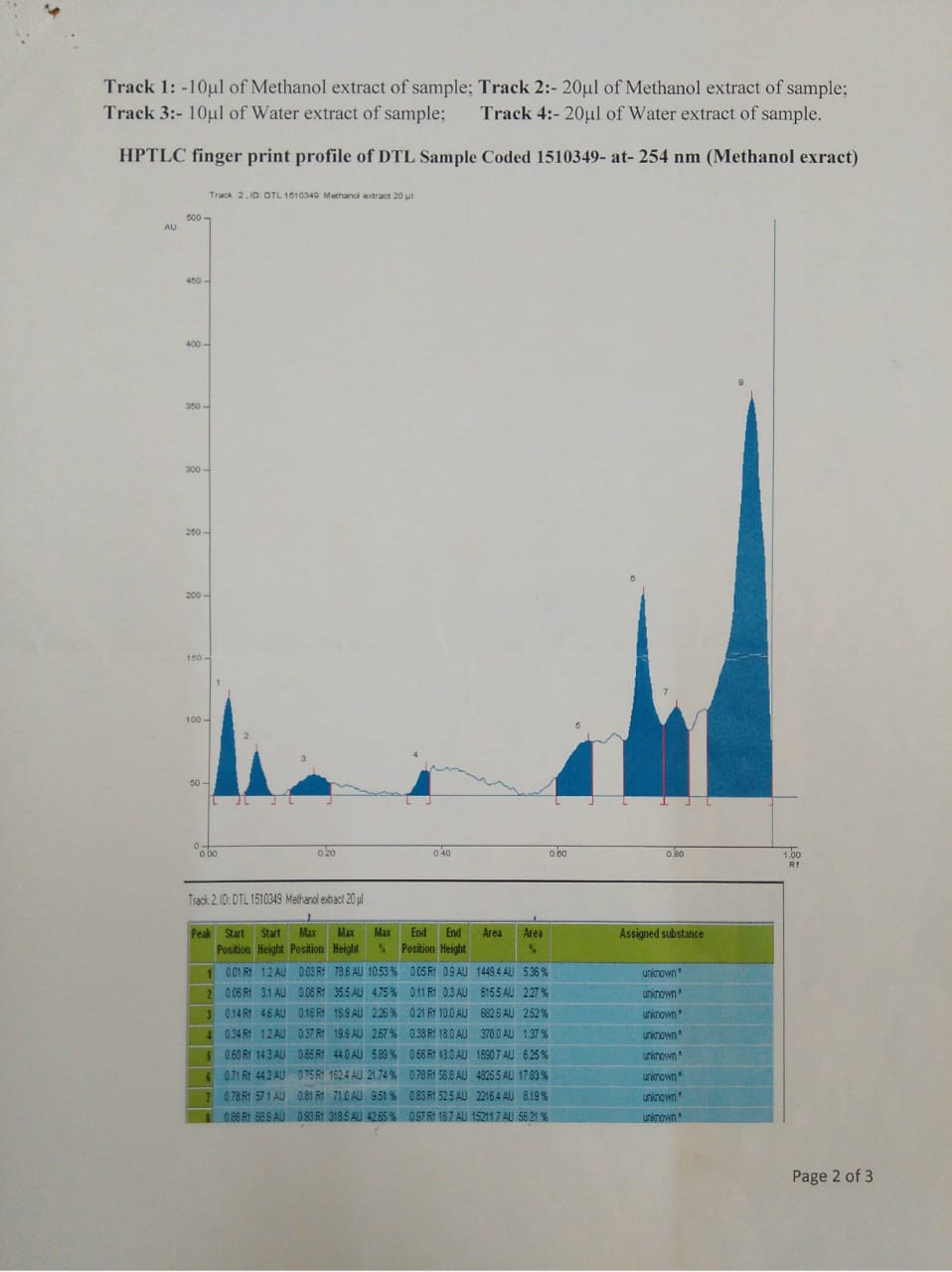


**Fig. S2. HPTLC densitogram of *Amorphophallus paeoniifolius* tuber**

(A) Methanol extract


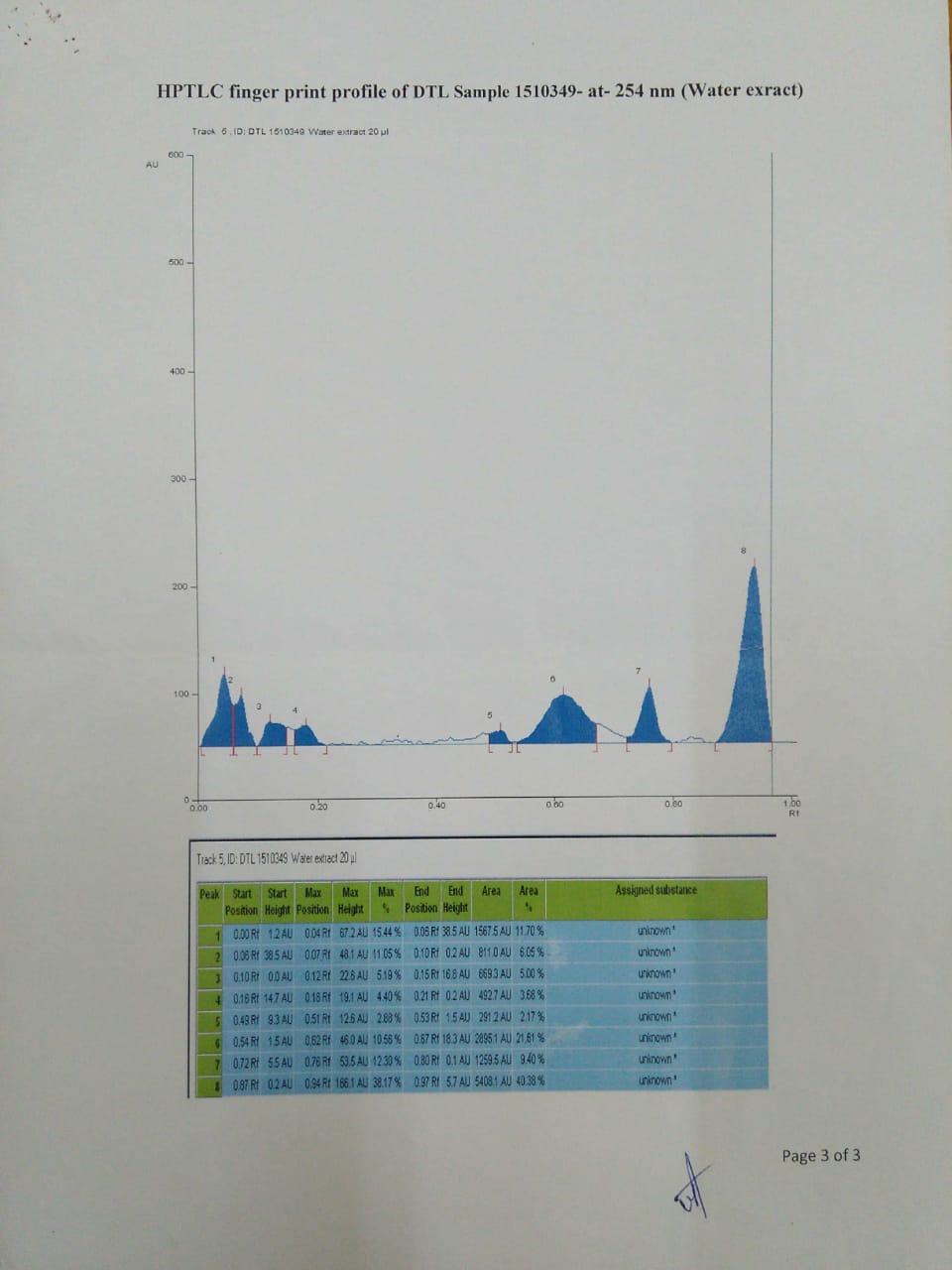


**Fig. S3. HPTLC densitogram of *Amorphophallus paeoniifolius* tuber**

(B) Aqueous extract
